# Investigation on law and economics of listed companies’ financing preference based on complex network theory

**DOI:** 10.1371/journal.pone.0173514

**Published:** 2017-03-16

**Authors:** Jian Yang, Shuying Bai, Zhao Qu, Hui Chang

**Affiliations:** 1 Law School, Tianjin University, Tianjin, China; 2 School of Foreign Languages and Literature, Tianjin University, Tianjin, China; Tianjin University of Technology, CHINA

## Abstract

In this paper, complex network theory is used to make time-series analysis of key indicators of governance structure and financing data. We analyze scientific listed companies’ governance data from 2010 to 2014 and divide them into groups in accordance with the similarity they share. Then we select sample companies to analyze their financing data and explore the influence of governance structure on financing decision and the financing preference they display. This paper reviews relevant laws and regulations of financing from the perspective of law and economics, then proposes reasonable suggestions to consummate the law for the purpose of regulating listed companies’ financing. The research provides a reference for making qualitative analysis on companies’ financing.

## Introduction

Financing is the main approach for listed companies to acquire financial support and achieve better achievement. Compared with traditional companies, scientific companies are less dependent on natural resources, but more on capital investment. Studying financing issues of scientific companies and analyzing financing difference caused by different governance structure can effectively circumvent the risk of corporate financing, provide basis for empirical analysis and promote the improvement of legal regulations specific to listed companies’ financing.

The present status of listed companies’ financing in China shows the following characteristics: first of all, internal capital accumulation is insufficient so the proportion of internal financing is relatively low. Secondly, external financing is the major financing source and the proportion of equity financing is large and the percentage of medium and long-term liabilities is less than that of current liabilities. Governance structure is one of the critical factors that affect financing decision making, therefore, to some extent, different governance structure of companies leads various financing decision. In this paper, governance structure is taken as the indicator to construct a complex network among selected listed companies. Most researches on listed companies’ financing issues are qualitative analyses or use statistical approaches to establish liner relation. Distinguished from other studies, this paper utilities complex network to analyze scientific companies’ financing preference and the influence of governance structure on financing, and then proposes practical suggestions to legal regulations from the aspect of regulating financing behaviors and optimizing governance structure.

According to existing literature, researches on listed companies financing analysis are mostly conducted through empirical analysis [1–7]. In previous research, complex network has been used to explore the cooperation preference among commercial banks, small enterprises and small loan companies [8]. By means of collecting scientific listed companies’ financing data and governance structure data, this paper utilizes complex network theory to study the influence of governance structure on financing decision. In this research, we divide sample companies into groups in accordance with the similarity of corresponding indicators, analyze prominent characteristics of each group and the influence of governance structure on financing, and furthermore, we compare the research results with current situation and conduct qualitative analysis. In the end, referring to related laws and administrative regulations, we make practical proposals to legal norms specific to regulating financing and optimizing governance structure, which is expected to bring new thoughts for financing research.

During recent years, the complex network theory has gone through remarkable progress and the study of complex network has become an interdisciplinary subject which arouses extensive attention from various disciplines [9–14]. In a complex network, the components are considered as nodes and edges represent the interactions between them. Therefore, the complex network is the mathematical representation of complex system. Time series analysis is a fundamental problem of continuing interest [15–17]. Quite recently, complex network analysis of time series elicits a great deal of interest from different research fields [18–27], and all of those have demonstrated their power in characterizing real complex systems from time series. Moreover, cooperative behaviors in social lives are widely studied [28–32]. In this research, complex network is used to analyze governance structure and financing data of scientific listed companies and group them according to the similarity. Based on grouping results, revealing the network topology is the key of the research.

## Materials and methods

Complex network is of vital importance in many natural systems for it can describe different kinds of complex systems which contain a large number of units with nodes and edges separately representing the component units and the interaction between nodes. In this research, a financing complex network has been established. The nodes stand for companies and the edge is determined by the strength of correlation between nodes, which means the similarity between companies. The correlation between two nodes is determined by the correlation between selected indicators. According to the method proposed by Prof. Gao etal. [33], here we illustrate how to use the strength of correlation between indicators to establish the edges and then construct the complex network. We use indicators of company’s governance structure and financing produce characteristic vector. For each pair of characteristic vectors, *T*_*i*_ and *T*_*j*_, the correlation coefficient can be written as:

$$C_{ij}=\frac{\sum_{k=1}^{m}\left[T_{i}(k)-\left\langle T_{i}\right\rangle \right]•\left[T_{j}(k)-\left\langle T_{j}\right\rangle \right]}{\sqrt{\sum_{k=1}^{m}{\left[T_{i}(k)-\left\langle T_{i}\right\rangle \right]}^{2}}•\sqrt{\sum_{k=1}^{m}{\left[T_{j}(k)-\left\langle T_{j}\right\rangle \right]}^{2}}}$$ (1)

M is the dimension of the characteristic vector and $\left\langle T_{j}\right\rangle =\sum_{k=1}^{m}T_{j}(k)/M$, $\left\langle T_{i}\right\rangle =\sum_{k=1}^{m}T_{i}(k)/M$. The elements *C*_*ij*_ are limited to the interval −1 ≤ *C*_*ij*_ ≤ 1 in which *C*_*ij*_, 0, and -1, respectively represent the perfect correlations, no correlation and perfect anti-correlations. C is a symmetric matrix and *C*_*ij*_ characterize the state of the connection between node i and j. Lastly, selecting an important threshold *r*_*c*_ and then adjacency matrix A can be formed by translating the correlation matrix C. The principles of conversion are as follow:

$$A_{ij}=\left\{ \begin{array}{l} 0,\left|c_{ij}\right|\leq r_{c}\\ 1,\left|c_{ij}\right|\geq r_{c} \end{array}\right.$$ (2)

It means if |*c*_*ij*_| ≥ *r*_*c*_, there will be an edge connecting node i and j, otherwise, there will be no edge. The complex network consists of all the nodes and edges and the corresponding topological structure can be revealed by the adjacency matrix A.

Newman (2004) proposed an idea of modularity Q. It is a quality function which is used to test whether the division of vertices into communities is meaningful and then community structure can be established. [34].

Supposing a network has n vertices. For a certain network divided into two parts, we set *s*_*i*_ = 1 if the vertex i is in part 1 and *s*_*i*_ = −1 if the vertex i is in part 2. And we annotate *A*_*ij*_ as the number of edges between vertices i and j. Although *A*_*ij*_ could be some large values in certain situations, it is 0 or 1 in most cases (there is always 0 or 1 edge between two vertices). But the expected number of edges between vertices i and j is, if edges are placed at random, where *k*_*i*_ and *k*_*j*_ are the degrees of the vertices and $m=\frac{1}{2}\sum_{i}k_{i}$ is the total number of edges in the network. The modularity can be written as

$$Q=\frac{1}{4m}\sum_{ij}(A_{ij}-\frac{k_{i}k_{j}}{2m})\;S_{i}S_{j}=\frac{1}{4m}S^{T}BS$$ (3)

where **s** is the vector whose elements are the *s*_*i*_. The leading factor of 1/4m issued for compatibility with the previous definition of modularity.

Then we can have a new real symmetric matrix **B** with elements

$$B_{ij}=A_{ij}-\frac{k_{i}k_{j}}{2m}$$ (4)

which is called the modularity matrix. We are focusing on the properties of this matrix. For now, we just get know of the elements of each the rows and columns are sum up to zero, therefore there is always an eigenvector (1,1, 1…) with eigenvalue zero.

Given Eq (3), we can let **s** as a linear combination of the normalized eigenvectors *u*_*i*_ of **B**, therefore $s=\sum_{i=1}^{n}a_{i}u_{i}$ with. After that we have

$$Q=\sum_{i}a_{i}u_{i}^{T}B\sum_{j}a_{j}u_{j}=\sum_{i=1}^{n}{\left(u_{i}^{T}•s\right)}^{2}\beta_{i}$$ (5)

where *β*_*i*_ is the eigenvalue of **B** mapping at eigenvector *u*_*i*_. Furthermore, we take away the leading factor of 1/4m for making it brevity.

The algorithm described above uses only of the signs of the elements of the leading eigenvector and the magnitudes convey information. Vertices related to elements of larger magnitude make large contributions to the modularity, and conversely for small ones. It shows that changing a vertex corresponding to a small magnitude element from group to another makes little difference to Q. Namely, elements’ magnitudes are a measurement of “how much” a vertex be a part of one community or another. That is to say, for vertices with elements near zero, they are on the borderline in communities. Therefore, the algorithm not only let us divides the vertices into groups, but let us changes them on a continuous scale of what degree they are part of one group or another. If a specific partition no longer produces within community edges and that would be expected by the random counterpart, Q = 0. Values other than 0 indicate deflection from randomness.

The detection of community structure in complex network is of vital importance and attracts much attention. It may be the best thought of as a data analysis method used to illustrate the structure of network datasets such as social network in this paper. In this paper, the approach for detecting the community structure is based on the approach proposed by Newman (2006) [35]. Normally, community structure approaches assume network of interest that naturally divides into subgroups and the purpose is to find out these groups. Besides, community structure approaches may particularly recognize the possibility that there exists no good division of the network, a result that is itself considered to be of interest for its influence on the topology of the network. In consideration of the freedom that we can choose the size of our groups of vertices, the greatest value of the coefficient ($u_{1}^{T}$) in this term can be achieved. Dividing the vertices according to the signs of the elements in **u**_**1**,_ following the principle that all vertices whose corresponding elements in **u**_**1**_ are positive go into one group and the rest in the other group. The algorthm is: we computing the major eigenvector of the modularity matrix and according to the signs of corresponding elements in this vector divide the vertices into groups.

The connections of resulting network are characterized by the threshold *r*_*c*_. The pairs of nodes with weak correlations will be connected even if *r*_*c*_ is extremely small. Under such situation, the physical content of correlations in time series will be hidden. The number of connections among nodes will reduce with the value of *r*_*c*_ increasing and more noise will be revealed. However, heretofore, there has not been a general method used for determining the critical threshold.

In this research, we can estimate a range of *r*_*c*_ where the structure of complex network is relatively stable and we use Q to select the critical threshold *r*_*c*_. Especially, though simulating a specific process of the complex network, a critical threshold *r*_*c*_ can be figured out. For example, decreasing the number of connections through increasing the value of *r*_*c*_ and keeping the modularity which determine network almost unchanged. According to Newman’ algorithm, when values of Q are greater than 0.3, it reflects significant community structure, and then we can find out the threshold [34].

## Application in complex network of financing activities

### Background knowledge

In a financing process, there are two kinds of financing: internal financing and external financing according to different sources of raised fund. Internal financing means investors utilizing their own savings. External financing is the way that investors make use of savings from others as their investment funds and debt-asset system is the major measure of external financing. Equity financing and debt financing are two ways of external financing. Equity financing refers to the way that companies raise funds from their shareholders or investors usually at start-up stage or to increase capital and share. Equity financing represents shareholders’ control to the ownership of companies. Debt financing is the way of raising capital from creditors through issuing bonus or getting bank credit. Debt financing represents creditors’ rights to the company. This paper selects equity financing and debt financing to analyze financing preference of scientific listed companies.

### Application in the financing analysis of scientific listed companies

In the research, the above method is used to reveal a real financing complex network. We select 45 scientific listed companies as sample companies and the data of governance structure and financing from 2010 to 2014, making comparative analysis to data of corresponding indicators, then construct the complex network. Listed companies represent nodes of the network. If two companies have high similarity in corresponding indicators, there is a link, otherwise, there is no link, and eventually the groups are formed to distinguish sample listed companies. Modularity changes in accordance with the change of threshold of each year, therefore, by referring to the data of each year and expected grouping results, we choose reasonable threshold and corresponding modularity to constitute groups.

According to the results, it is found that with the increasing of threshold, the modularity of governance structure rises first and then falls down and reaches the peak when threshold is 0.21. In the figure of the year 2010 and 2014, modularity keeps maximum value when threshold is in the range from 0.21 to 0.24, therefore, we choose 0.21 as the modularity of governance structure complex network and the corresponding threshold values of each year are Q = 0.3902, Q = 0.3958, Q = 0.4810, Q = 0.5000 and Q = 0.4795.

In the modularity distribution of financing structure, the modularity value overall increases with the rise of threshold and reaches the peak when the threshold is in the range from 0.95 to 0.99. Therefore, the threshold and modularity of financing structure complex network for each year are r = 0.95, Q = 0.4908; r = 0.99, Q = 0.6626; r = 0.96, Q = 0.3457; r = 0.99, Q = 0.7414; r = 0.96, Q = 0.3848. All modularity values are larger than 0.3 so it can form a stable network structure. According to the modularity, all nodes were grouped and the complex network was constructed.

## Results and discussion

### The influence of governance structure on listed companies financing

#### Influence of management on financing decision

a. The Influence of Structure and Size of Board of Directors on Financing Structure

In *Company Law of the People's Republic of China* [36], article 114 regulates that the board of the directors of a company may decide that a member of the board of directors shall serve concurrently as the manager. Therefore, whether the chairman of the board serves concurrently as the manager, which is the structure of board of directors, affects the financing decision in some degree. Specifically, when the chairman of the board serves concurrently as the manager, the control of company is firmly concentrated and the problem of insider control will be serious, therefore, under such circumstance, management prefer equity financing to reduce the agency cost. When the chairman of the board is not the general manager, board of directors prefers debt financing as the restriction and supervision to managers as well as to reduce the agency cost. The size of board of directors also decides the decision making. In *Company Law of the People's Republic of China*, Article 44 regulates that a limited liability company shall have a board of director of three to 13 members. Article 108 regulates that a company limited by shares shall have a board of directors of five to 19 members. When choosing equity financing, it is easier to impact the benefit distribution of large shareholders. Therefore, if the size of board of director is larger, the divergence in terms of the coordination and communication among different investors will be greater and it will be more difficult to reach a united opinion. Under such condition, the benefit from equity financing will be reduced. If the company chose debt financing, the stock rights of shareholders which represent their control of company would not be weakened and their status remain unchanged, so it is easier to make an agreement on benefit distribution among different shareholders and the decision-making cost of debt financing is lower than that of equity financing. Taking the above factors together, listed companies with large size of board of directors tend to debt financing to reduce the contradiction between different investors and the cost of decision making; those with smaller size are apt to equity financing.

b. The Influence of Independent Director System on Financing Structure

The independent director system refers to independent directors coming into the board on behalf of small shareholders and they play their role in supervision, nomination and payment assessment. *The Guiding Opinions on the Establishment of Systems of Independent Directors by Listed Companies* [37] regulates that each listed company in China should engage suitable persons as independent directors in accordance with the requirements. At least one of the independent directors should be a professional accountant. At least one-third of the members in the board of directors should be independent directors. One of independent directors’ duties is protecting and maintaining shareholders’ interests especially for small and medium shareholders. Therefore, introducing the independent director system is to reduce the possibilities of irregularities of listed companies. One of independent directors’ governance goals in making financing decisions process is to make restriction to the management and funds operation through debt financing, therefore, debt financing is preferred by independent directors. On the one hand, debt financing introduces creditors to restrain management and large shareholders and then reduce the cost of internal governance; on the other hand, rigid restrictions of creditor’s rights can avoid company operators’ excessively free use of funds which may lead to bankruptcy and threaten the reputation of independent directors.

c. Influence of Management Shareholding on Financing Structure

According to principal-agent theory, increasing managers’ shareholding may make the interest of top management and shareholders become consistent, reduce the cost of external equity agency cost and better achieve motivation and restriction to operators. Once shareholding, senior management do not want their control of company to be diluted so they support debt financing to improve the asset-liability ratio. When the proportion of senior management’s shareholding is high, managers have no worry in the dilution of their shareholding, so they prefer equity financing based on the consideration of cost.

d. The Influence of the Size of Supervisory Board on Financing Structure

In *Company Law of the People's Republic of China*, article 51 regulates that a limited liability company shall have a board of supervisors, which shall have no fewer than three members. A limited liability company with comparatively few shareholders and comparative small in scale may have one to two supervisors instead of a board of supervisors. The board of supervisors shall include an appropriate ratio of representatives of the company’s staff and workers, in which the ratio shall not be lower than one third. The responsibilities of supervisory board are supervision and restriction. Reasonable size of supervisory board and board members perform their duties to make the company apply appropriate financing strategy and use financial leverage to create more profit for the company. Hence, reasonable size of supervisory board is conductive to supervise the board of directors making better financing decisions.

#### The influence of ownership situation on financing decision

Ownership concentration refers to the quantitative index of equity concentration or equity dispersion due to the different share proportion of all shareholders. It is a representative indicator of ownership situation. Ownership concentration is usually represented by the sum of shareholding ratio of the top five or top ten shareholders (CR5 or CR 10). Currently, ownership of listed companies in China is mostly highly concentrated and state-owned shares are in large proportion. Companies in high ownership concentration tend to equity financing considering that the control of company is highly concentrated in one or a few shareholders, the stake holders not only get most profit of companies’ production and operation, but also bear most of the loss and risks when companies fail in operation and face loss. Under such circumstances, the profit pattern is characterized by high-risk and high-yield, therefore, investors would choose equity financing to spread the investment risk and reduce venture capital cost. At the same time, due to the high ownership concentration, equity financing will not waver or weaken big shareholders’ control of the company. Furthermore, to maximize investment income, big shareholders will utilize all means and methods actively to supervise and manage operators’ behaviors in daily operation.

### Grouping results and analysis

**Fig 1:**
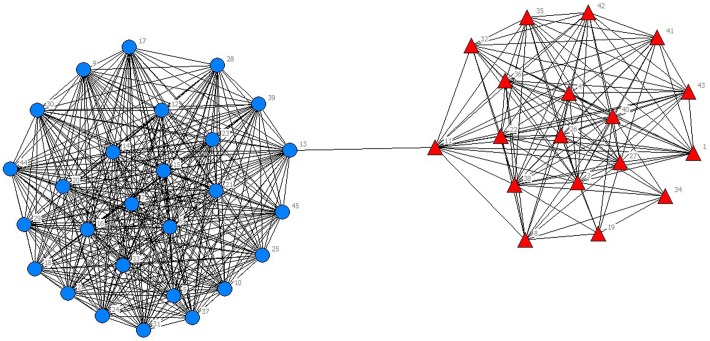
Complex Network of Governance Structure in 2010.

**Fig 2:**
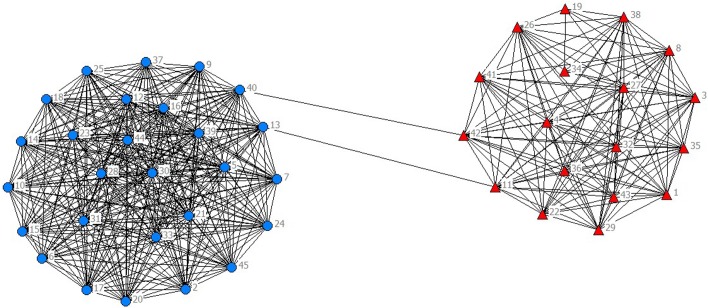
Complex Network of Governance Structure in 2011.

**Fig 3:**
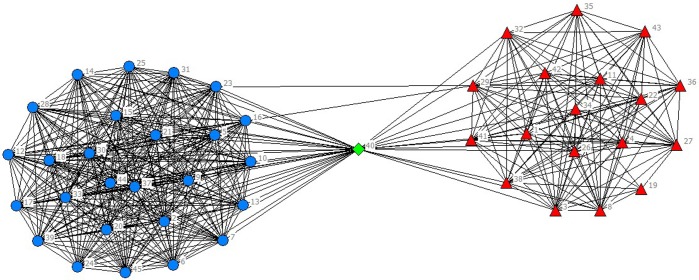
Complex Network of Governance Structure in 2012.

**Fig 4:**
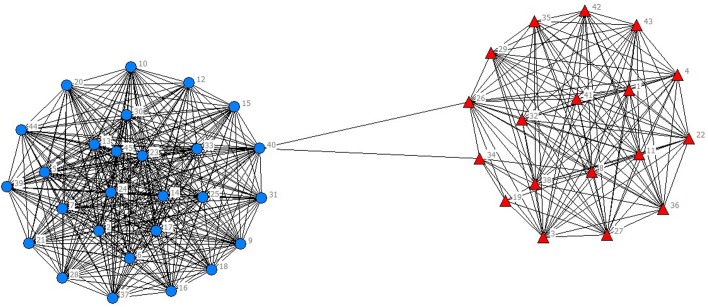
Complex Network of Governance Structure in 2013.

**Fig 5:**
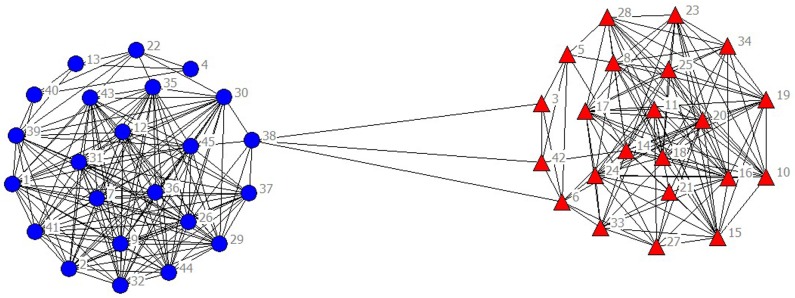
Complex Network of Governance Structure in 2014.

**Fig 6:**
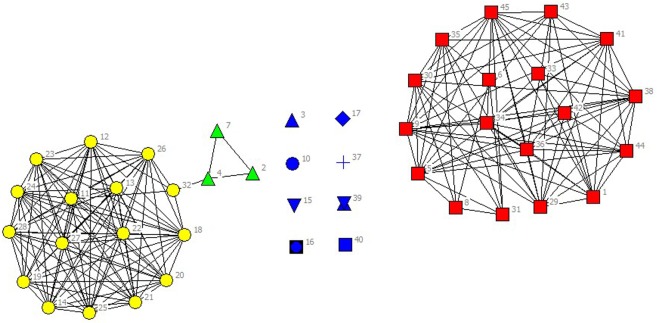
Complex Network of Financing Structure in 2010.

**Fig 7:**
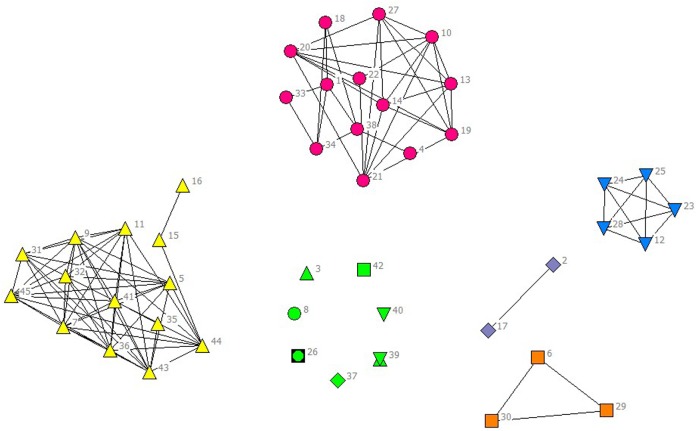
Complex Network of Financing Structure in 2011.

**Fig 8:**
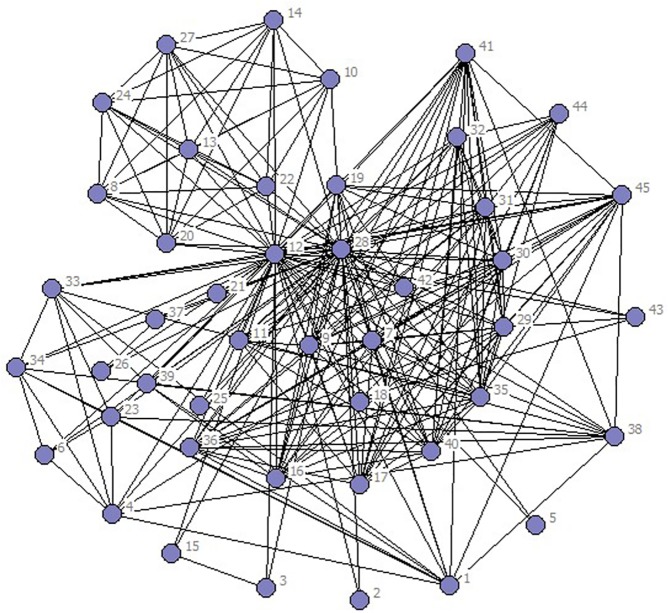
Complex Network of Financing Structure in 2012.

**Fig 9:**
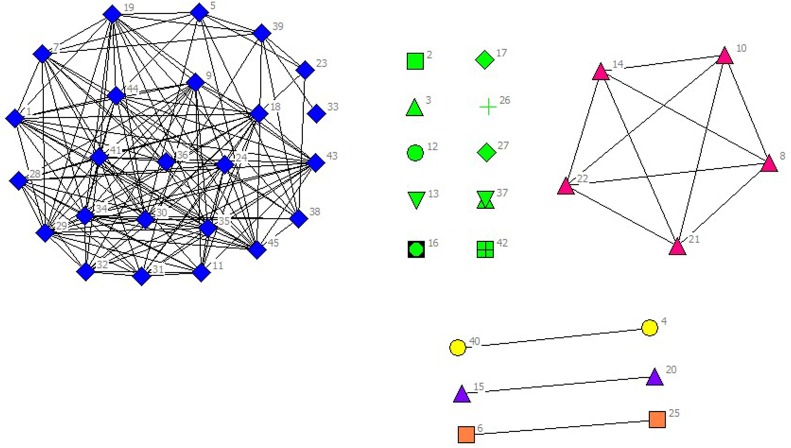
Complex Network of Financing Structure in 2013.

**Fig 10:**
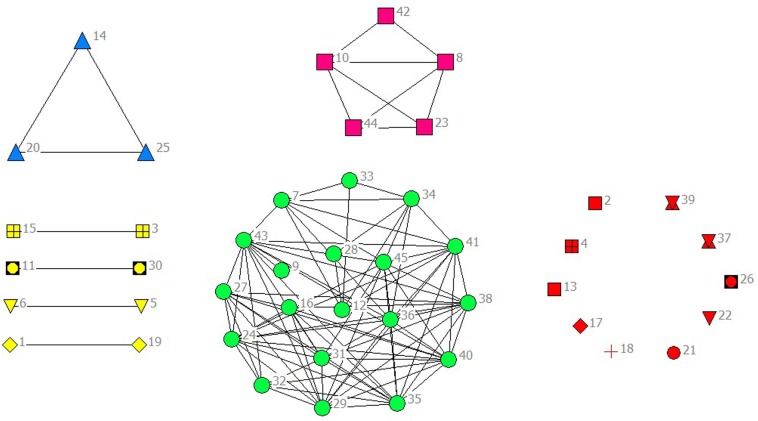
Complex Network of Financing Structure in 2014.

In the selected data from 2010 to 2014, scientific listed companies’ governance structure is in high similarity; therefore, the grouping results are almost stable. In real situations, listed companies would change the equity ratio of next year or adjust the management settings according to indicators of previous year such as operating performance, future development goals, so the grouping results of financing structure of each year have slight difference. The results are as showed in Figs 1–10.

For company No.40 (stock code 600718), the size of board is six, the number of independent director of 2010 is five, three for 2011to 2013 and seven for 2014. The proportion of shares that the board of directors owns is higher than 70%. The proportion owned by supervisory board is less than 1% and by senior management is less than 1.5%. The ownership concentration is high, reaching 63.4%. The proportion of the largest shareholder is over 17%, which is higher than the second-largest shareholder.

In the first group, listed company No. 13 (stock code 002405) has nine members in the board, with five independent directors and three members in the supervisory board. Senior executives’ shareholding is less than 10%. The actual controller of the company is China Aerospace Science and Technology Corporation. Its ownership concentration is 60.1%, and the largest shareholder owns 24% shares.

For company No.16 (stock code 300025), the board size is four; president holds a concurrent post as general manager. It has three independent directors, and three members in board of supervisors. Ownership concentration is close to 53.9%. The shareholding of the largest shareholder is 22.2%, which is much higher than the second-largest shareholder.

In the second group, in respect of company No.11 (stock code 002373), the size of board is six, the number of independent director is three or four. The proportion of shares that the board of directors owns is high. The supervisory board has three members and its proportion of shareholding is 0. The ownership concentration is high, reaching 72.3%. The actual controller is individual. The proportion of the largest shareholder is 26.6%, which is higher than the second-largest shareholder.

Company No.26 (stock code 300096) has three members in the board. The proportion for the board is 41.58%. The size of supervisory board is three and the proportion of shareholding is 0. The proportion for senior management is higher than 5%. The ownership concentration is high, closing to 60%. The actual controller is individual. The largest shareholder is over 35%, which is higher than the second-largest shareholder.

The above grouping results reflect and present the governance situation of nowadays listed companies in China now: state ownership is high, president holding a concurrent post as general manager is much more common; executive directors own significant stakes; independent directors scale are quite different; independent director system is still imperfect, executive ownership gap is big; and ownership concentration is overall high.

Despite the differences between governance structures, sample companies’ financing data from 2010 to 2014 generally shows significant equity financing preference. Therefore, the grouping results of financing shows the feature of “high concentration and small scatter” which makes the equity financing become prominent. Specifically, asset-liability ratio is generally lower; in the total amount of financing. The proportion of equity financing share is significantly higher than that of loans and issued bonds. We select typical companies to illustrate and analyze financing decision of listed companies under governance structure.

First of all, the funding data of company No.16 shows that both equity financing and debt financing are included as financing methods, with low rates of assets and liabilities. The sources of debt financing are loans, without bond. The influences of corporate governance structure on financing decision are: if the board size is small, there are fewer conflicts of interest among shareholders and they tend to equity financing; second, when president holds a concurrent post as general manager and the shareholding of the largest shareholder is much higher than that of the second-largest shareholder, it will easily lead to internal control problems. Both executives holding and ownership concentration are high, so it is unnecessary to worry about equity dilution of shareholders’ control over the company. In consideration of financing costs and obtainable benefits, shareholders tend to equity financing, however, under such circumstance, independent directors and board of supervisors are hard to play their role.

In respect of company No. 13, the financial data shows annual debt to asset ratio from 2010 to 2014 is less than 15%. Financing mostly comes from the equity financing and in debt financing, short-term loans owns a large proportion. The influence of corporate governance structure on financing decision is: the board size is large while executives’ holding is in low proportion, so executives tend to avoid equity dilution. In order to avoid interest conflicts, the board of directors prefers credit financing. However, this company is state-owned listed companies, the actual controller is the state, and state holding is the largest part which reflects the characteristic of most state-owned listed companies in China and it may lead to “autocratic” domination and management tendency and administrative operation means. Corporate governance with strong executive factors is not conducive to make appropriate financial decisions, and at the same time, owning to the high concentration of ownership, large shareholders are more likely to choose the equity financing in order to diversify financing risk and to reduce financing cost.

For company No.17 with stock code 300036, it has six directors in the board with three independent directors, and three supervisors in the board of supervisors. The board of directors owns more than 30% shares for three consecutive years. The supervisory board holds less than 1% shares and executives hold more than 17% shares. The company is controlled by individuals with an average ownership concentration of 37.5%. The largest shareholder owns over 15% shares and the second largest shareholder owns 7%. The company’s financing figure shows that: from 2010 to 2014, asset-liability ratio increased and the average ratio is 22.37%. Most of the financing comes from equity financing. Debt financing includes both short-term loans and long-term loans, in which the latter accounts for a major, and annually there are goodwill loans. The impact of corporate governance is reflected as follows: slightly smaller board size and higher proportion of shareholding of the board result in the board of directors owning most right on enterprise decision making. It is difficult for the board of supervisors with a small shareholding to play their role. Executives do not worry about dilution of their stake caused by equity financing because of the high proportion of shares. Therefore, they prefer equity financing. The company is actually controlled by individuals, combined with a higher proportion of shareholding of the largest shareholder and the board, for this reason, financing decisions are mainly controlled by large shareholders. The company's asset-liability ratio exceeds 20%, but it still prefers equity financing in which there are both long-term and short-term loans, so the company is in the development stage with a great demand for funds, including both demand for quick turnaround and long-term investment. The company ownership concentration is moderate, which is conducive to scientific and rational financing decisions.

The financing data of company No.40 reflects that, from 2010 to 2014, the asset-liability ratio increased and reached 42.06% in 2014. Equity financing is the major source of financing. Short-term loans are major parts of debt financing, which indicates that the company needs large amount of capital for turnover. The company had goodwill loans each year and issued bonds in 2011. The influences of governance structure on financing making can be summarized as: the proportion of executive directors and shareholding is high, therefore the control to the company is under the board of directors; the size of board of supervisors is small and the proportion is low, so the function of supervision cannot works; the high ownership concentration ensures large shareholders do not worry about the dilution of their stake, and for the sake of agent cost, therefore equity financing is preferred.

In addition, the financing data of a small number of listed companies reflects a higher debt ratio where current liability is a major part, which means that the company prefers debt financing. However, these companies are still in a minority. The majority of listed companies display a significant preference of equity financing, which is consistent with the overall financial situation of listed companies in China now.

### Analysis of equity financing preference

#### Reasons for equity financing preference

Equity financing satisfies companies’ long-term demand for funds and is mainly used for long-term investment. The funds raised by equity financing will be a part of ownership interest which is not allowed to be reduced unless the company faces bankruptcy. Equity financing owns advantages that the interest is not required to pay and dividend distribution can be set according to companies’ operation.

The main reason of preferring equity financing preference is the low cost of financing. The cost of equity financing consists of dividend and issue expenses. It is common that listed companies do not allocate the dividend on shares and the payout ratio is low. Even if the dividend is allocated, cash distribution is the least and issuing bonus and stock transfer are more common, therefore the payout of stock dividend is extremely low. Moreover, the constraints of issuing bonds are greater than that of issuing stock. To sum up, equity financing is better preferred by listed companies compared with debt financing.

In addition, in the aspect of corporate governance, there are problems of insider control, the imperfection of internal governance mechanism and the lack of effective external governance mechanism, which go against the optimization of financing structure.

#### The way of equity financing and legal norm

Currently, specific ways of equity financing are:(1) The allotment and sale of shares to the original shareholders (hereinafter referred to as allotment of shares); (2) Publicly raising shares towards against unspecified objects (i.e. making additional issuance); (3) Publicly issuing convertible corporate bonds; and (4) Private offering of stocks.

Excessive equity financing will result in a series of problems. Firstly, it will inevitably lead to the dilution of equity and if the business has no corresponding development and growth, the net income per share will reduce and ultimately affect the benefit of the company. Moreover, equity financing that over a certain reasonable limit is neither conductive to the long-term development of stock market nor favorable to the normal operation of financial system. In financing market, equity financing, bond financing and credit financing are basic financing methods and they should be promoted and developed under the guidance of market operating patterns and fully play their advantages. Listed companies’ preference of equity financing will bring about the imbalance of China’s capital market and eventually influence the secure operation of financial system.

According to the practice and reasons of listed companies’ equity financing preference, this paper proposes the following suggestions to related legal regulations. First, the supervision policy of government should appropriately raise the requirements for equity financing. For example, in the article of *Administrative Measures for the Issuance of Securities by Listed Companies* [38], raising the requirement of allotment, which is the weighted average yield rates of net asset for the latest 3 years. In addition, stock dividends is an important component of equity financing cost, and the phenomenon of low dividends yield, no dividends payout and malignant dividends of listed companies are serious. To restrain such phenomenon, government should make clear policy restraints to dividends method and payout ratio, as well as making specific regulations to dividends. For example, *Decisions on Amending Some Provisions on Cash Dividends on Listed Companies* [39] have mended the dividend rate. “The profits which it has accumulatively distributed in cash in the recent 3 years shall not be less than 30% of the average annual distributable profits realized in the recent 3 years.” The relation between additional issuance to specific objects and cash dividends should be mentioned and supervisions to listed companies’ dividend system should be risen to legal level.

Equity crowd funding raises as a new method of private financing provides more opportunities for ordinary investors to participate venture capital. The remarkable features of crowd funding are publicity, uncertainty and a number of investors with small amount money. At present, the legal status of China’s equity crowd funding is unclear and corresponding legal supervisions are deficient, which increases the legal risks. Therefore, the legislation is required as soon as possible. For example, referring to Article 87 of *Securities Investment Fund Law of the People’s Republic of China* [40] which is the regulation towards qualified investors of non-publicly offered funds and setting the standards for qualified investors from the aspects of asset size or income level, corresponding risk identification ability and risk tolerance and investment upper limit. Besides, defining specific obligations of investors and increasing the disclosure responsibility of issuers.

In terms of governance structure, the equity concentration is high and controlling shareholder's stake is too high which causes the serious problems of insider control. The structure of listed companies’ board of directors is still unreasonable. It is common that the chairman of the board holds a concurrent post as general manager and the proportion of executive directors is too high. The independent director system is still imperfect and the independence of the board is still a problem that has been widely questioned. Under such circumstance, the board would be manipulated by company’s major shareholders and cannot play its function. To solve such problems, firstly, it is recommended that the proportion of executive directors in board of directors should be limited. The improvement of independent director system will better represent and safeguard the interest of minority shareholders. Although *Company Law of the People's Republic of China* has stipulated that shareholder shall not abuse their rights, the regulation has wide meaning with low operability and has not established corresponding accountability mechanism for major shareholders’ abuse of rights. Therefore, it is recommended to establish a connected transaction accountability mechanism. Increasing the liability of controlling shareholders who damage the interests of the company, not only in terms of civil liability, but also in terms of criminal and administrative responsibility. The voice of minority shareholders in decision making should be heard. Furthermore, to protect the rights of minority shareholders, cumulative voting system and proxy of voting rights should be developed and improved.

In accordance with *Company Law of the People's Republic of China*, the board of supervisors shall include an appropriate ratio of representatives of the company’s staff and workers, in which the ratio shall not be lower than one third. Shareholder supervisors are responsible for the nominee, however, nominee in general is the large shareholder who masters the control of the company, and employees in board of supervisors are under the leadership of the board of directors and managers. Therefore, the composition of the board of supervisors decides that it is difficult for supervisors to implement effective supervision to the directors who occupy the position of majority shareholding. Moreover, it is easy to lead to the formalization of supervision. In this paper, we make the proposal that relevant laws should make clear and specific regulations in view of the above problems. For example, restricting the proportion of executive directors in the board, improving the independent director system on order that independent director will better represent and protect the interests of minority shareholders. Besides, to avoid the formalization of supervision, the law shall clearly define the composition of board of supervision such as regulating the proportion of staff and workers shall not be less than 50% and comprehensively considering the performance of supervisory duties.

## Conclusion

This paper establishes complex networks of listed companies’ financing and governance structure by taking scientific listed companies as examples. By means of analyzing characteristics of each network group, we explore prominent features of governance and analyze the influence of governance structure on financing decision. We figure out that the result is consistent with realistic situation that under the influence of governance structure, most scientific listed companies have evident equity financing preference. To conduct further research, we refer to relevant regulations of existing law and put forward optimization suggestions to the provisions concerning equity financing and cooperate governance structure that are not specified in the law. Complex network time series analysis has become an important topic and has been applies in many research fields [18–27,41]. The method proposed in this paper belongs to complex network time series analysis. Other than study on legal analysis, we expect the method has its place in many other research fields.
